# Fractionation versus Adaptation for Compensation of Target Volume Changes during Online Adaptive Radiotherapy for Bladder Cancer: Answers from a Prospective Registry

**DOI:** 10.3390/cancers15204933

**Published:** 2023-10-11

**Authors:** Christoph Pöttgen, Christian Hoffmann, Thomas Gauler, Maja Guberina, Nika Guberina, Toke Ringbaek, Alina Santiago Garcia, Ulrich Krafft, Boris Hadaschik, Aymane Khouya, Martin Stuschke

**Affiliations:** 1 Department of Radiotherapy, University of Duisburg-Essen, Hufelandstr. 55, 45147 Essen, Germany; 2 Department of Urology, University of Duisburg-Essen, 45147 Essen, Germanyboris.hadaschik@uk-essen.de (B.H.); 3 German Cancer Consortium (DKTK), Partner Site University Hospital Essen, University of Duisburg-Essen, 45147 Essen, Germany

**Keywords:** online adaptive radiotherapy, bladder cancer, dose accumulation, equivalent uniform dose

## Abstract

**Simple Summary:**

Online adaptive radiotherapy (ART) allows adaptation of the dose distribution to the anatomy captured with pre-adaptation imaging. Since ART is time-consuming, intra-fraction deformations can occur, antagonizing the advantages of ART. In this study from a prospective registry, the effects of intra-fraction deformations of the clinical target volume (CTV) on the equivalent uniform dose (EUD_CTV_) of focal radiotherapy of invasive bladder cancer were evaluated. Using margins of 5–10 mm around CTV, intra-fraction CTV-deformations reduced the effect of ART, lowering the 10th percentile of EUD_CTV_ values per fraction from 101.1% to 63.2% of the prescribed dose. A strong fractionation effect was demonstrated, making the EUD_CTV_ for a series of dose fractions insensitive to EUD_CTV_-declines per fractions. Two dose accumulation methods showed that EUD_CTV_ values were >92.5% for all series of dose fractions, which could be explained by the small size and the spatial variations of cold spots. Our data showed that ART for each dose fraction is unnecessary.

**Abstract:**

Online adaptive radiotherapy (ART) allows adaptation of the dose distribution to the anatomy captured by with pre-adaptation imaging. ART is time-consuming, and thus intra-fractional deformations can occur. This prospective registry study analyzed the effects of intra-fraction deformations of clinical target volume (CTV) on the equivalent uniform dose (EUD_CTV_) of focal bladder cancer radiotherapy. Using margins of 5–10 mm around CTV on pre-adaptation imaging, intra-fraction CTV-deformations found in a second imaging study reduced the 10th percentile of EUD_CTV_ values per fraction from 101.1% to 63.2% of the prescribed dose. Dose accumulation across fractions of a series was determined with deformable-image registration and worst-case dose accumulation that maximizes the correlation of cold spots. A strong fractionation effect was demonstrated—the EUD_CTV_ was above 95% and 92.5% as determined by the two abovementioned accumulation methods, respectively, for all series of dose fractions. A comparison of both methods showed that the fractionation effect caused the EUD_CTV_ of a series to be insensitive to EUD_CTV_-declines per dose fraction, and this could be explained by the small size and spatial variations of cold spots. Therefore, ART for each dose fraction is unnecessary, and selective ART for fractions with large inter-fractional deformations alone is sufficient for maintaining a high EUD_CTV_ for a radiotherapy series.

## 1. Introduction

Bladder cancer is one of the few tumor entities, where planning target volume (PTV) margins >10 mm are used around the clinical target volume (CTV) for standard image-guided radiotherapy (IGRT) to cover the CTV with the prescribed dose from a treatment plan scheduled for a series of dose fractions in the presence of anatomical deformations between fractions [1]. Bladder cancer ranks as the 10th most common diagnosed cancer worldwide with a four times higher incidence in men than in women [2]. About 25% of bladder cancers are muscle-invasive cancers [3]. The median age of patients with muscle-invasive bladder cancer at diagnosis is about 75 years [4]. Radiotherapy within a trimodality treatment including transurethral resection of the bladder tumor, radiotherapy, and chemotherapy is an accepted treatment option for patients with muscle-invasive bladder cancer for patients preferring an alternative to radical cystectomy and for those who are medically unfit for surgery [5]. An analysis in the National Cancer Data Base revealed a similar survival of patients treated with trimodality treatment in comparison to cystectomy for bladder invasive [6]. Trimodality treatment is rarely used in Germany for patients with muscle-invasive bladder cancer; only 57 patients received definitive radiochemotherapy in comparison to 698 patients receiving cystectomy for locally advanced bladder cancers in the year 2021 in the certified Uro-oncology centers [7]. In England, however, radical radiotherapy was performed more often than cystectomies in the year 2016 for muscle-invasive bladder cancer [4].

Larger PTV margins—with the aim of adequate coverage of the clinical target volume—increase the irradiated bladder volume that is related to bladder toxicity, as shown for high-dose radiotherapy of prostate cancer [8]. Online adaptive radiotherapy is recently available either on an MRI-Linac- or a cone beam CT (CBCT)-based system [9]. With this technology, the adaptation of the plan on a prefraction imaging study is possible but time-consuming, ranging from 10 min to more than 30 min for a CBCT- and MRI-based therapy system [10,11]. It is a promising new technology that allows dose escalation with the prospect of better tumor control [12]. Plan adaption requires the delineation of organs at risk and the CTV on the prefraction imaging study, as well as optimization of the dose distribution according to clinical goals on this anatomy. Within this time frame, profound deformations and volume changes of the bladder can occur [13]. Volumetric imaging on a linear accelerator is fast and can be performed within 30 s with a CBCT and deformations of the anatomy after plan adaptation can be captured rapidly before treatment; this is highly recommended, as important anatomic changes can occur within the treatment timeframe, affecting the delivered effective dose [14,15]. However, the effect of site-specific anatomical deformations during adaption on the accumulated doses from a series has not yet been evaluated sufficiently, nor the influencing parameters that are responsible. In general, ART is introduced for the whole treatment series and not selective on worse fractions with image-guided radiotherapy (IGRT), which can lead to unnecessarily prolonged treatment times and inefficient use of this rather limited resource. Therefore, it would be of great advantage to predict shortly after the first on-couch imaging whether online ART will have an advantage over treatment with the scheduled plan optimized before the start of the treatment series [16].

The equivalent uniform dose (EUD) is a parameter used to quantify the biological effect of an inhomogeneous dose distribution on tumor or normal tissues [17]. For tumors, different model parameters were used with the generalized EUD model from a = −20 to a = −5, where a = −20 is suitable for rather aggressive tumors [18]. The generalized EUD model at a = −20 showed a very high correlation with a model based on linear quadratic cell survival for lung cancer using alpha/beta coefficients for inter-fraction recovery of 4 Gy [19]. This alpha/beta value is also within the range of values estimated for bladder cancer [20]. 

Deformable image registration (DIR)-based dose accumulation is an important constituent of adaptive radiotherapy [16]. The displacement vector field generated with DIR is used to wrap the three-dimensional dose distribution from the source to the reference CT. However, beneath spatial uncertainties of displacement vector field generated with DIR, dosimetric inaccuracies can occur. For dose accumulation on the reference CT, the dose-volume histogram for the CTV in the reference CT with the wrapped adapted dose distribution of the day should be the same as the dose-volume histogram for the deformed CTV of the day in the prefraction imaging study with the adapted dose distribution. This consistency however might depend on the deformation algorithm used and the voxel sizes of the images [21,22]. In dependence on the DIR-based dose accumulation algorithm used, the minimum doses in the deformed CTV of the day might not be preserved during the wrapping of the dose distribution on the planning CT [21,23]. The minimum dose in the tumor, however, critically determines the EUD at the model parameters used [19].

This study aims to quantify the effects of intra-fractional deformations during the adaptation time needed for focal adaptive radiotherapy of bladder cancer with margins of ≤10 mm around the CTV, as well as the effects of dose fractionation on the EUD for the accumulated dose distribution based on data from consecutive patients from a prospective registry treated with CBCT-guided ART. Worst-case dose accumulation was introduced directly from the dose-volume histograms to the dose fractions of a series, and compared with DIR-based dose accumulation. The effect of spatial variation of the locations of the cold spots in the CTV was analyzed by reordering the links between DVH used for dose accumulation.

## 2. Materials and Methods

### 2.1. Patients

Consecutive patients treated with definitive online-adaptive radiotherapy at the ETHOS^®^ therapy System (Varian, Palo Alto, CA, USA) were analyzed. All patients provided written informed consent and were registered on a prospective institutional clinical registry. The study was approved by the Ethics Committee of the University of Duisburg-Essen (18-8364-BO, 23-11380-BO). The study was conducted according to the Declaration of Helsinki. All patients had invasive bladder cancer and were initially discussed in an interdisciplinary tumor board of urologists, radiation oncologists, and medical oncologists. Trimodality bladder conserving treatment was offered to patients with significant risk factors for radical cystectomy and to patients who denied cystectomy as necessary definitive surgery. 

All patients underwent pretreatment stages including physical examination, routine hematologic and biochemical tests, cystoscopy with histological biopsy, and CT/MRI and/or PET/CT of the thorax and abdomen. Before radiotherapy, all patients received a maximal transurethral resection of the bladder tumor (TURBT). All patients were offered concurrent chemotherapy according to their comorbidity, with cisplatin as the preferred option or gemcitabine as an alternative for patients non-eligible for cisplatin [24,25]. 

Between 12/2021 and 3/2023, nine consecutive male patients were treated with ART; patients had a median age of 67 years (range: 54–86 years) and a median Charlson Comorbidity score of 5 points (range: 3–6 points). The ECOG performance status was 0 in five patients, 1 in three patients, and 2 in one patient. All patients had invasive high grade urothelial carcinomas. Tumor location was centered to the dorsal or lateral walls in 78% of patients, the trigone of bladder in 11%, and the dome of the bladder in 11% of the patients. None of the patients had clinically detectable lymph node metastases. 

### 2.2. Radiotherapy—Planning and Treatment

Patients were irradiated after TURBT with a tumor-directed focal target volume of up to total doses of 64–66 Gy—within the conventional total dose range [25]. Planning CT studies were performed with a half-full bladder in order to spare a non-involved contralateral wall with focal ART. Delineation of the macroscopic gross tumor volume (GTV) was based on information on initial cystoscopic and CT findings. After TURBT, the tumor bed was marked with Lipiodol injection into the bladder wall in two of the patients, and the planning CT was performed on the same day within 3 h after Lipiodol injection as described by Pos et al. [26]. For focal bladder tumor radiotherapy, the clinical target volume (CTV) was constructed from the pretreatment GTV with an axial and a cranio-caudal margin of 5–10 mm. The planning target volume (PTV) included the CTV with individualized 5–10 mm margins, to compensate for intra-fractional deformations. Larger PTV margins can severely alter the ability to spare the non-involved bladder wall with focal radiotherapy. For example, a bladder volume of 200 mL corresponds to an equivalent sphere with a radius of 3.6 cm. If the CTV overlaps with one-half of the bladder wall and assumes a spherical bladder shape, a PTV margin of 15 mm would lead to a 32% smaller bladder wall segment that is not overlapping with the PTV than a margin of 5 mm. The maximum faction of the bladder wall that can be spared by ART decreases accordingly.

The bladder wall was contoured as a bladder contour with an inner margin of 3 mm [27]. Focal radiotherapy was either delivered for the whole series or as a boost after whole bladder irradiation of up to a dose of 41.4 Gy in 23 fractions. Elective lymph node irradiation was not planned. 

Online-adaptive radiotherapy was performed on the ETHOS^®^ therapy system. At the start of a dose fraction, an initial cone beam-CT (CBCT1) was performed. With the aid of artificial intelligence algorithms, contouring of the bladder, rectum, and prostate was performed using the system and reviewed by a board-certified radiation oncologist. Corrections were made if necessary. Thereafter, the system was used to deform CTV from planning CT prior to the start of the radiation series onto CBCT1 using these structures. The resulting CTV was reviewed and modified by the attending radiation oncologist when necessary. The PTV was derived from the CTV by adding individualized margins from 5 to 10 mm in different directions. The system generated a synthetic CT with appropriate Hounsfield Unit values for photon dose calculation by deforming the planning CT onto the daily anatomy as imaged by the CBCT1. For each fraction, two plans were created on the synthetic CT—the adapted and the scheduled plan. The adapted plan was optimized on the anatomy of the day according to the initially defined clinical goals (CTV: D_99%_ ≥ 98%, Bladder [focal therapy]: D_mean_ ≤ 55%, and Rectum: V_30_% ≤ 35%). In four of the treatment series, a volumetric modulated arc treatment technique was chosen, which takes about 13 min of planning calculation time and in the remaining time, a 12-fixed field intensity-modulated radiotherapy technique was chosen that took about 3, 4 min for plan optimization [28]. 

The scheduled plan was a recomputation of the initial plan on the daily anatomy. After reviewing both plans, one of them was selected for treatment. Subsequently, a second cone beam CT (CBCT2) was acquired to detect intra-fractional anatomical changes or patient movements in order to perform an additional image guidance procedure. If necessary, a translational couch shift for local adjustment was performed according to CBCT2.

### 2.3. Follow-Up

After treatment completion, patients were planned for re-evaluation every three months for the first two years, then every six months for the next three years, and then annually including physical examination, hematologic and biochemical tests such as cystoscopy with biopsy of suspect lesions and CTs of thorax and abdomen. Toxicity was documented according to the common terminology criteria of adverse events (CTCAE v5.0).

### 2.4. Bladder, Rectum and CTV Delineation in CBCT2

The bladder and rectum were contoured offline in CBCT2 using an artificial intelligence-based auto-segmentation algorithm (MIM ProtégéAI^®^, MIM Software Inc., Cleveland, OH, USA, version 1.1.3). The resulting structures were reviewed by two experienced radiation oncologists and adapted when necessary. Two deformable image registration (DIR) algorithms (MIM Maestro^®^, MIM Software Inc., Cleveland, OH, USA, version 7.3.2) were employed to propagate the CTV from CBCT1 to CBCT2 after co-registering both imaging studies using the volumetric modulated arc treatment translation vector determined during the online match. A bladder and rectum contour-based DIR (DIR1) and a hybrid algorithm that utilized both intensity information and the bladder contour (DIR2) were used. Both DIR are free-form deformable registration algorithms [29]. The approved CTV in CBCT1 was deformed onto CBTCT2 using DIR1, and the result was reviewed. If deemed necessary, corrections were made by an experienced radiation oncologist while viewing the CTV in CBCT1 side by side. In a second step, another radiation oncologist inspected the new CTV in CBCT2. This inspection involved viewing the superposed CTV in CBCT2 corrected by the first reviewer, as well as the CTV in CBCT2 deformed by both DIR1 and DIR2. The approved CTV in CBCT1 was presented side by side. Additional changes were made and the final CTV was consented by both reviewers. 

### 2.5. Equivalent Uniform Dose

The Equivalent Uniform Dose (EUD) is a concept to characterize the effect of a heterogeneous dose distribution on organs at risk or the tumor. The EUD is the homogeneous dose that results in the same biological effect as the non-uniform dose distribution if delivered to the entire target volume or organ at risk. The generalized EUD was calculated in this study using Matlab (version R2022a) by applying the Lesbesque-norm for the dose distribution, with a tissue-specific parameter “a” according to the equation:

$$EUD={\left(\frac{1}{N}*\sum_{i=1}^{N}D_{i}^{a}\right)}^{\frac{1}{a}}$$

where *N* is the total number of voxels in the respective volume, *i* is the index of each voxel in the set of voxels making up the respective volume, and D_i_ is the dose in voxel *i* [17]. All EUD values were normalized to the prescribed dose (nEUD). For tumors, we used the parameter value a = −20, adequate for rather aggressive tumors [18]. The EUD model was successful in describing the effects of non-uniform dose distributions from brachytherapy or stereotactic radiotherapy on tumors such as lung or prostate cancer, and the effects of homogeneous dose distributions on normal tissues such as bladder [30,31]. Effective uniform doses were calculated for the bladder wall outside the PTV using model parameters of a = 1 and a = 8, which were within the range of values used in previous studies [32,33,34].

### 2.6. Deformable Image Registration-Based Dose Accumulation

An automatic workflow was created in the MIM^®^ environment to perform deformable image registration (DIR)—based dose accumulation. A rigid fusion between CBCT1 or CBCT2 and the planning CT was performed based on the isocenter positions derived from each fraction’s scheduled plan. This fusion was the starting point of a contour-based DIR between CBCT1 or CBCT2 and the planning CT. The CTV contours were used for the generation of this deformation. Dose distributions calculated on the synthetic CTs were then wrapped on the planning CT using this DIR. Voxel-wise dose accumulation was performed at an isotropic resolution of 1 mm^3^/voxel.

### 2.7. Worst-Case Dose Accumulation

In addition to dose accumulation using elastic deformation of the CTV in CBCT2 onto the reference CTV in the planning CT and adding dose in the corresponding voxels in the reference CTV, an independent procedure of worst-case dose accumulation was established, relying on the dose-volume histogram data from the different dose fractions for the CTV in CBCT2 alone. Differential dose-volume histograms of the CTV in CBCT2 for the different dose fractions were obtained with MIM^®^ using the adaptive plan. Radiation doses for each fraction were normalized to the prescribed dose and dose bins of 0.1% were used. The relative volumes in the dose bins contained multiples from 10^−5^ percent of the CTV, denoting 10^7^ subvoxels of the CTV, which represented the precision limit of relative volume calculation. Datasets were created in SAS containing 10^7^ rows that represented the 10^7^ subvoxels from the CTV at the volume resolution limit ordered according to dose with increasing row order numbers from the lowest to the highest dose. Different voxels of the same dose bin had consecutive but different row numbers. For worst-case dose accumulation, the doses from the different dose fractions were added in the subvoxels of the same order number from the lowest to the highest number so that cold spots from all fractions fall onto each other. This extreme spatial correlation between the cold spots of the dose distributions from the different dose fractions leads to the lowest nEUD_CTV2_ for the accumulated dose distribution. This follows directly from the concave dose response using the generalized uniform dose concept with an a-norm parameter of a = −20 for aggressive tumors [35].

In addition to worst-case dose accumulation, the effect of cold spot redistribution between dose fractions was studied using two methods. According to method 1, random permutation of the order numbers of a fixed percentage x of the coldest voxels per fraction was performed and doses were accumulated in voxels according to the new order numbers across the fractions. Method 2 used random permutation of a given percentage y of the coldest subvoxels over a 5 mm thick surface rim of the CTV for all dose fractions except the dose fraction with the lowest nEUD_CTV_ value. The order of the voxels of the dose fraction with the lowest nEUD_CTV_ remained unchanged. The percentages of subvoxels in the outer 5 mm rim of the CTV were calculated for the CTV in the planning CT and ranged from 27.7% to 39.3% using the volume of the CTV and spherical geometry. It was assumed, that the outer rim of the CTV contained the coldest voxels of the CTV. The percentage x of the coldest subvoxels redistributed between fractions was increased until the accumulated dose across the respective adaptive series exceeded 95% of the prescribed dose. Random redistribution was repeated at least three times at each percentage X point of the coldest voxels redistributed between fractions in the datasets (see Figure 4b–e).

### 2.8. PTV Margin Calculation

A commonly applied margin recipe, according to van Herk (2000), was used to calculate the margin at a considered point of the CTV surface to ensure that, in at least 95% of dose fractions, the point is within this margin for at least 90% of the patients [36]. Hereby, normal distributions of the random deviations and systematic errors were assumed from series to series, perpendicular to the CTV surface of the target volume at the considered point. Random errors were combined with the dose fall-off at the PTV margins. The inward–outward deformation of the margin of the CTV is a motion in one dimension. We chose the global Hausdorff point as a point for consideration at the surface of the CTV. This point represents the maximum distance observed across all the fractions of a treatment series between the considered CTV and the reference CTV, i.e., between the CTV of the respective dose fraction in CBCT1 and the planning CT for IGRT, or between CBCT2 and CBCT1 of the same fraction for ART. As the mean deviation at the global Hausdorff point across all patients and fractions was not 0 mm, this overall mean deformation was also included in the margin recipe [37]. The global Hausdorff points were found at the bladder dome for all patients, thus the outward direction was the superior direction and the dose fall-off was described by a Gaussian distribution with a sigma of 3.2 mm. The 95% confidence limits for the 95th percentile of the superior–inferior deviation of the global Hausdorff point between fractions was calculated using order statistics.

In addition, the PTV margin was obtained by a second margin growing method. This involved expanding the CTV in the reference CT anisotropically in the +/−x, +/−y, and +/−z directions of the image datasets from all dose fractions and all treatment series. The expansion was carried out in 1 mm increments so that the deformed CTV in CBCT1 for IGRT or in CBCT2 for ART is fully and tightly encompassed by the expanded volume in ≥95% of dose fractions [38,39].

### 2.9. Statistics

Nonparametric statistics were used to compare the empirical distribution functions with the different treatment series (proc npar1way, SAS version 15.1, SAS Institute Inc., Cary, NC, USA). Logistic regression and regression on ranks or continuous values were performed with SAS procedures proc logistic and proc GLM. Multiple testing was considered in interpreting *p*-values.

## 3. Results

Between 12/2021 and 3/2023, 137 dose fractions were delivered with online ART to focal bladder tumor target volumes in nine male patients. For each treatment series, 8 to 36 fractions were delivered. Four patients received focal bladder tumor radiotherapy alone while five patients received focal radiotherapy as a boost series after whole bladder radiotherapy. Target volumes in the planning CT ranged from 80 to 356 mL, with a median of 218 mL. Individualized PTV margins ranged from 5 to 10 mm in the cranial and anterior directions, 5 to 7 mm in the latero-lateral direction, and 5 to 9 mm in the caudal direction.

**Figure 1:**
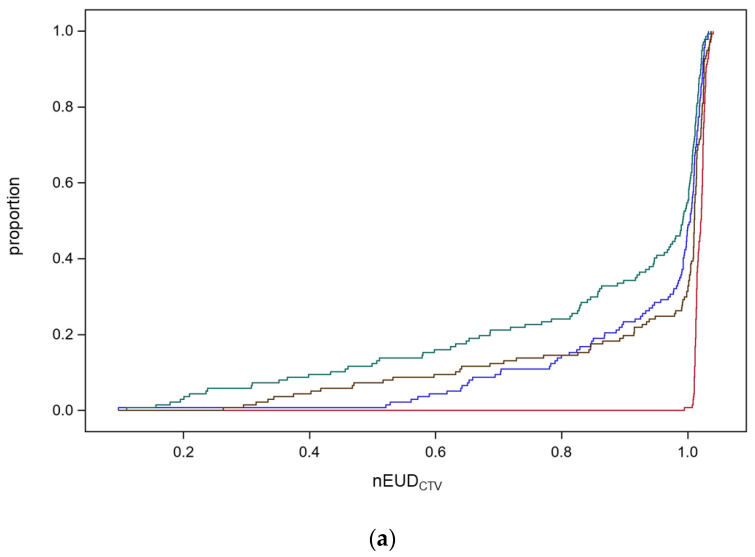

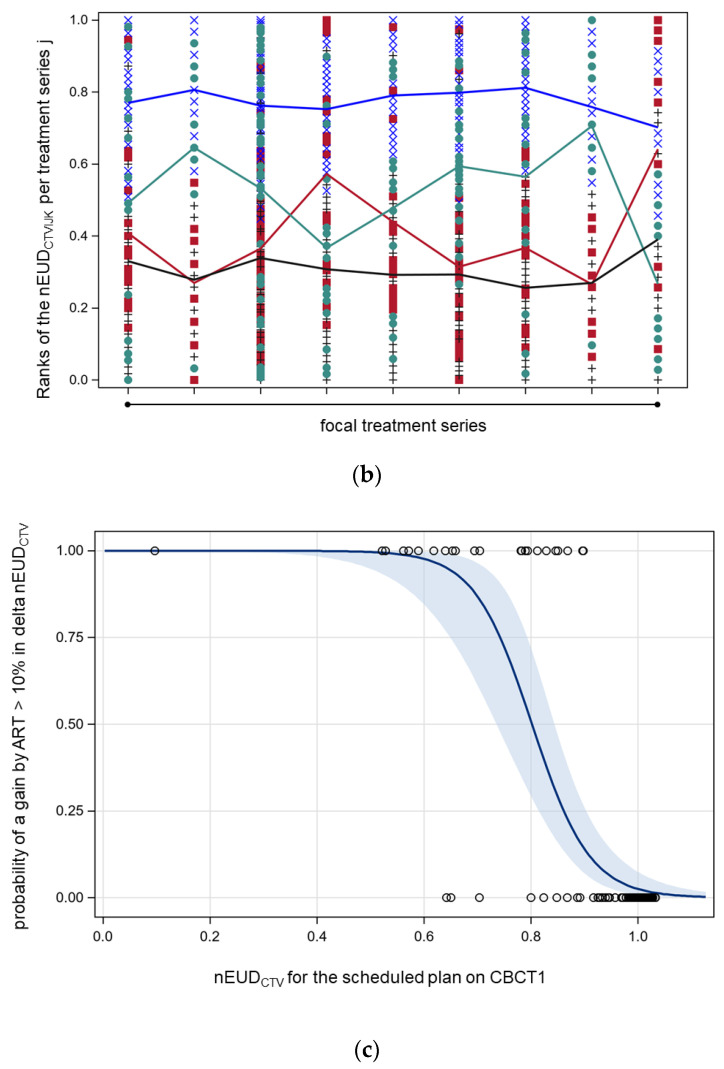
(**a**) Empirical distribution functions (EDFs) of the normalized equivalent uniform doses for the CTV (nEUD_CTV_) for the nine focal bladder tumor radiation series using the adaptive plan for the CTV on CBCT1 (red) or CBCT2 (brown), or the scheduled plan on CBCT1 (blue) or CBCT2 (green). The narrow band EDF for the adaptive plan on CBCT1 degrades towards CBCT2. (**b**) Normalized ranks of the nEUD_CTV_ within each focal treatment series according to the treatment modes using the adaptive plan on the anatomical scenarios in CBCT1 (blue circles) or CBCT2 (green rhombus), or the scheduled plan on CBCT1 (red crosses) or CBCT2 (grey triangles). On average, the adaptive plan on CBCT1 resulted in the highest ranks, followed by the adaptive plan on CBCT2, the scheduled plan on CBCT1, and the scheduled plan on CBCT2. (**c**) The dependence of the probability of a gain by ART in comparison to IGRT with an increase in nEUD_CTV_ ≥ 10% in comparison to IGRT (delta EUD_CTV_). Solid line: predicted probability with 95% confidence limits.

The empirical distribution functions of the EUD values across the 137 dose fractions from the nine focal bladder tumor irradiation series are shown in Figure 1a for the different treatment modes, delivering the scheduled plan on the anatomy captured by CBCT1 (CBCT1_sch), delivering the adapted plan on the anatomy of CBCT1 (CBCT1_ada), the scheduled plan on CBCT2 (CBCT2_sch), and the scheduled plan on CBCT2 (CBCT2_ada).

CBCT1_sch reflects the scenario of image-guided radiotherapy (IGRT); CBCT2_ada is the most precise estimate of the delivered dose by adaptive radiotherapy (ART) with a time delay between CBCT1 and dose delivery caused by plan adaptation. The changes in anatomy during this time interval are captured by CBCT2. The median, the 25th, and 10th percentile of empirical distribution function for the nEUD_CTV_ values and their 95% confidence intervals were 102.1% (95% CI: 101.7–102.3%), 101.3% (95% CI: 101.2–101.4%), and 101.1% (95% CI: 101.0–101.2%) for the online adaptive mode on CBCT1; 101.1% (95% CI: 100.9–101.3%), 97.9% (95% CI: 86.5–100.5%), and 63.2% (95% CI: 41.8–84.3%) for the online adaptive mode on CBCT2; as well as 100.4% (95% CI: 99.6–100.9%), 92.8% (95% CI: 84.8–98.2%) and 70.3% (95% CI: 64.0–82.4%) for the IGRT scenario. There was a significant decrease in the nEUD_CTV_ values and a broadening of the scale of the nEUD_CTV_ distribution for the online adaptive plans calculated for the CTV in CBCT2 in comparison to the CTV in CBCT1 due to anatomical deformations during the adaptation with median times of 18.8 min (5th–95th percentile, 10.9–30.7 min). Both nEUD_CTV_ distribution functions differed significantly in location and scale at *p* < 0.0001. Location was compared using the nonparametric Kruskal–Wallis test, which is based on a one-way analysis of variance statistics for Wilcoxon scores. The scale was compared by the non-parametric Mood test after subtracting the corresponding class median from each observation before performing the analysis. Mood scores were computed as the square of the difference between the observation rank and the average rank. The proportion of dose fractions with nEUD_CTV_ values below 70% and 80% of the prescribed dose was 9.5% and 14.6% with IGRT and 12.4% and 14.6%, respectively, with ART using the anatomic scenario on CBCT2 or 0.0% and 0.0% on CBCT1. There was also a significant decrease in the nEUD_CTV_ values from ART to IGRT per fraction with a median of −0.9% (95% CI: −1.4–−0.2%, *p* = 0.024, Kruskal–Wallis test). In addition, we observed a slight decrease in the nEUD_CTV_ for IGRT with the scheduled plan for the CTV in CBCT2 compared with CBCT1 due to deformations that took place during adaptation with a median of −0.5% (95% CI: −1.0%–−0.2%, *p* < 0.0001, Kruskal–Wallis test). There were significant differences in the distributions of nEUD_CTV_ values between treatment series (*p* = 0.0005, Kruskal–Wallis test). The 10th percentile of nEUD_CTV_ values was smaller than 90% in seven of the nine series.

For comparison of the four different treatment modes within each of the nine focal bladder tumor radiotherapy series, the nEUD_CTV_ values from the same series were ranked according to their size, and the ranks were normalized to an interval of [0, 1] per series. Comparing all series, the adaptive plan on CBCT1 resulted in the highest ranks on average, followed by the adaptive plan on CBCT2, the scheduled plan on CBCT1, and the scheduled plan on CBCT2 (analysis of variance on ranks, *p* < 0.01 for all pairwise, *t*-test, and Sidak correction for multiple comparisons between treatment modes were applied) [40]. Figure 1b depicts the interaction plot with a horizontal position representing the different treatment series. Different marker styles and colors represent the modes of radiotherapy. The mean ranks of the different treatment modes were connected by lines. The overall least squares means of the ranks of the adaptive plans were 77.2% and 51.6% on CBCT1 and CBCT2, respectively, and the scheduled plans were 40.5% and 30.6%, respectively. The standard errors of the least squares means were ±2.2%; however, there was significant heterogeneity from series to series. The adaptive plan on CBCT2 was better in seven and worse in two series in comparison to IGRT. The gain of adaptation per dose fraction was the largest for fractions with worse nEUD_CTV_ using IGRT. In addition, we compared a selective use strategy of ART with either IGRT or ART for all fractions. Performing ART selectively for dose fractions with nEUD values < 93% with IGRT was sufficient to eliminate the significant differences in ranked nEUD values between the selective ART strategy and ART (*p* > 0.05, *t*-test). Using this strategy, ART was only needed in 25.5% of dose fractions. 

In summary, the distribution of the nEUDCTv values degraded towards lower doses form CBCT1 to CBCT2 for both the scheduled and adaptive plans due to intra-fraction deformations. On average, ART on CBCT2 ranked slightly better than the scheduled plan on CBTC1 among the treatment series. 

Figure 1c shows that the probability of an increase in nEUD_CTV_ using ART instead of IGRT increases by 10% (delta_nEUD_CTV_ > 10%), with a decrease of nEUD_CTV_ with IGRT. Using logistic regression, there was as steep relation between such an increase and nEUD_CTV_ with IGRT (*p* < 0.0001, chi^2^ test) and the probability of such a gain by IGRT was 50% at an nEUD_CTV_ by IGRT of 80.2% (95% CI: 75.3–85.1%). This was a consequence of the only weak correlation of nEUD_CTV_ values using ART or IGRT (Spearman rank correlation coefficient of 0.33 (95% CI: 0.17–0.47)). The agreement of observing nEUD_CTV_ values < 80% at the same fraction with IGRT and ART was purely by chance (overall kappa-coefficient −0.04 and 95% CI: −0.13–0.04) with significant heterogeneity between treatment series (chi^2^ test for equal kappa coefficients, *p* = 0.0002). The median nEUD_CTV_ with IGRT for dose fractions with a delta_nEUD_CTV_ ≥ 10%, ≥20%, and ≥30% using ART instead of IGRT was 0.70, 0.65, and 0.59, respectively. The proportion of dose fractions per series with nEUD_CTV_ values below 80% using IGRT ranged from 0% for four series to 45% for one series. 

In summary, a substantial gain by ART can only be expected for the 15% of fractions with worse nEUD_CTV_ values < 80% by IGRT. 

**Figure 2:**
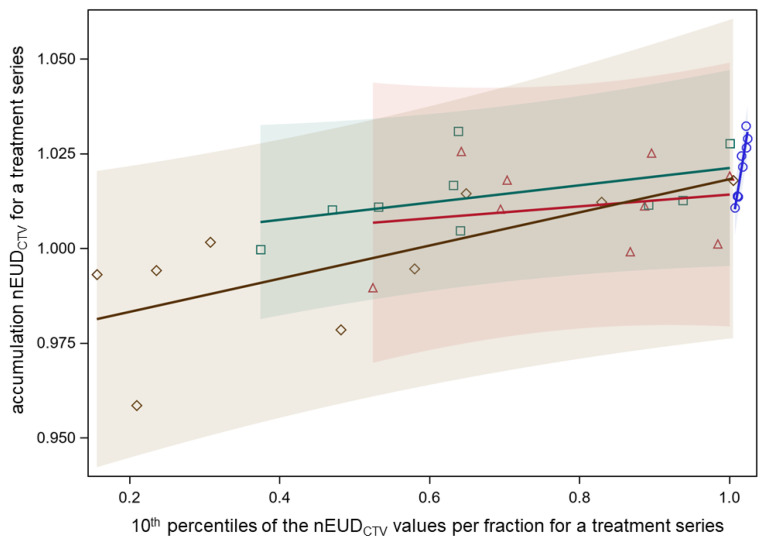
Dependence of nEUD_CTV_ for the accumulated dose distribution per treatment series on the 10th percentile of the nEUDi_CTV_ values for the dose fractions of the respective series. Separate relations fitted for the adaptive plans on CBCT1 (blue circles), the scheduled plans on CBCT1 (red triangles), the adapted plans on CBCT2 (green squares), and the scheduled plans on CBCT2 (brown diamonds). Colored transparent confidence bands for an individual prediction are shown (overall mean slope of 0.033 ± 0.010, and not significantly dependent on the treatment mode).

The nEUD_CTV_ for the DIR-based accumulated dose distribution across a treatment series is the most important parameter determining the effectiveness of the delivered radiotherapy series. Figure 2 shows the accumulated nEUD_CTV_ values using deformable image registration in dependence on the 10th percentiles of the distribution of the nEUDi_CTV_ values per dose fraction for the nine focal radiotherapy series. The data using both IGRT and ART as well as the adaptive plan on CBCT1 and the scheduled plan on CBCT2 are shown. The dependency of accumulated nEUD_CTV_ values on the 10th percentiles was rather shallow with an overall mean slope of 0.033 ± 0.010 and was not significantly dependent on the treatment mode (*p* = 0.23, F-test). Similar dependencies existed on the 25th and 50th percentiles with mean slopes of 0.046 ± 0.011 and 0.101 ± 0.026 (all *p* < 0.005, F-tests), respectively. The differences in the accumulated nEUD_CTV_ for a treatment series between IGRT and ART were small, ranging from −2.6% to 2.3% of the prescribed dose, with a median increase with ART of 0.3%. Selective ART for dose fractions with nEUD values < 93% had accumulated nEUD values in the middle between IGRT and ART for all fractions.

Therefore, the DIR-based accumulated dose distributions with ART across a treatment series were rather insensitive to the observed decreases in the nEUD_CTV_ values for single-dose fractions from CBCT1 to CBCT2 due to intra-fraction anatomical deformations during the time needed for dose adaptation. A similar effect of DIR-based dose accumulation across the fractions of a treatment series was observed for IGRT, mitigating the decrease of the nEUD_CTV_ values for single-dose fractions from inter-fraction deformations in bladder cancer. 

**Figure 3:**
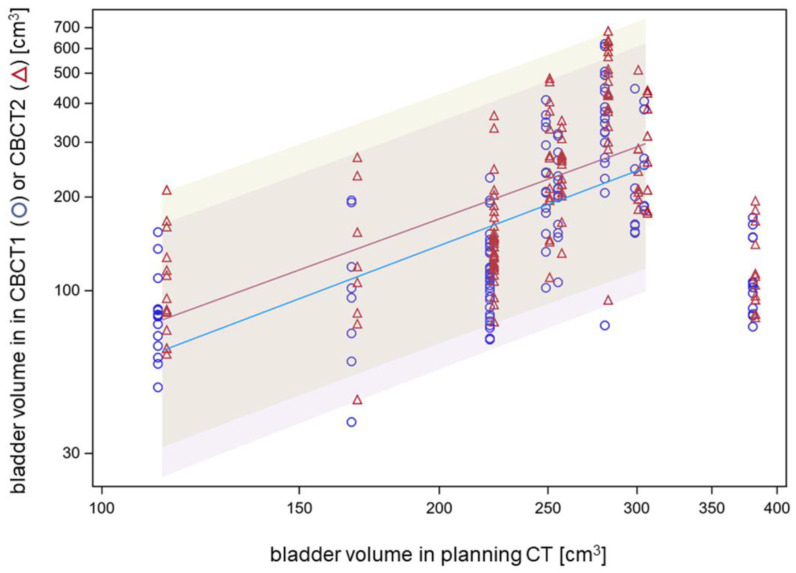
Association between bladder volumes in CBCT1 or CBCT2 and bladder volume in the planning CT on a logarithmic scale.

Patients were treated with a full or half-full bladder for focal bladder cancer irradiation in order to spare the contralateral (non-involved) bladder wall by the achievable dose gradients. Bladder volumes in the planning CT ranged between 113 and 381 mL, with a median of 256 mL. Across all patients, there was a strong association between logarithms of the bladder volume in CBCT1 or CBCT2 and the logarithm of the bladder volume in the planning CT for bladder volumes up to 310 mL. The slope of the linear fit was 1.34 ± 0.10 (*p* < 0.0001, F-test). The geometric mean ratio between bladder volume in CBCT2 and bladder volume in planning CT was 77%, and the respective ratio with bladder volume in CBCT2 was 92% (Figure 3). One patient was planned with a full bladder at 381 mL but this volume could not be reproduced and required prolonged online adaptation. The median increase in bladder volume from CBCT1 to CBCT2 was 27.5 cm^3^ (5th–95th percentile: −6.5–115.2 cm^3^), which was significantly dependent on the patient with median values from 5.9 cm^3^ to 71.9 cm^3^ (*p* < 0.0001, Kruskal–Wallis test). The inter-fractional deviation of the bladder volume on CBCT1 from the bladder volume in the planning CT was in the median of −72.2 cm^3^. 

Therefore, bladder volumes around 250–300 mL can be recommended for the bladder wall-sparing ART of bladder cancer. The PTV margin should anticipate the increase in bladder volume during the time needed for dose adaptation. Using spherical geometry, an increase in bladder volume from 250 cm^3^ to 350 cm^3^ during adaption means an increase in bladder radius by 4.6 mm. 

**Figure 4:**
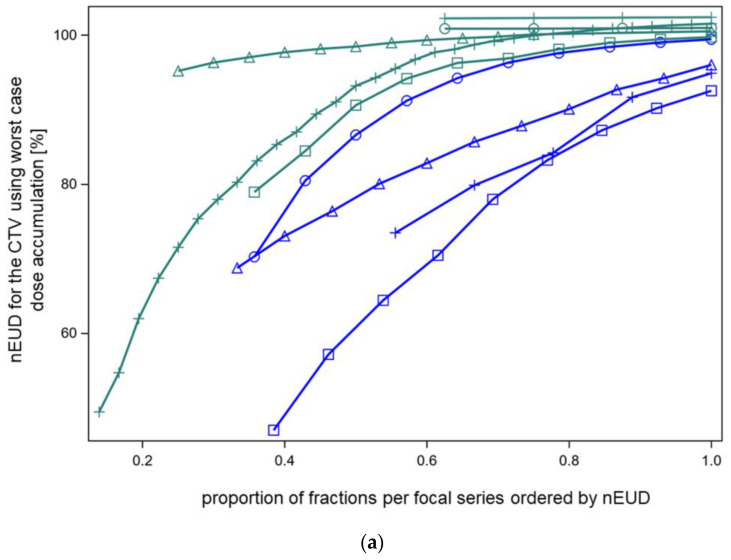

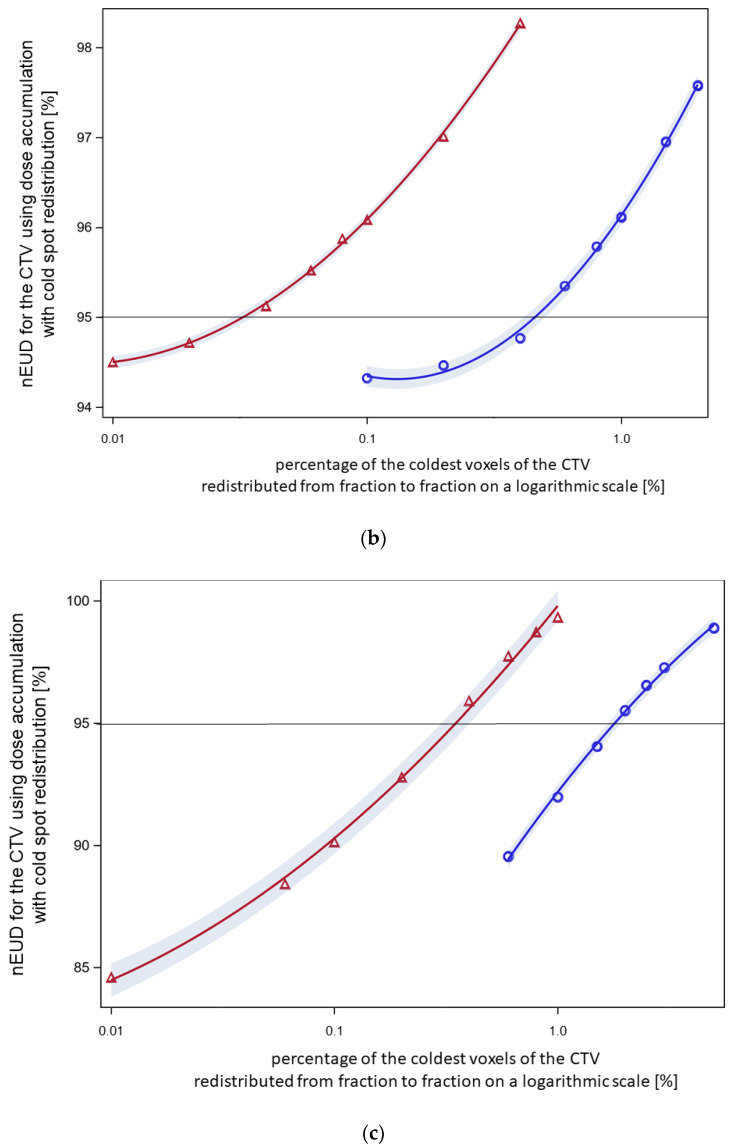

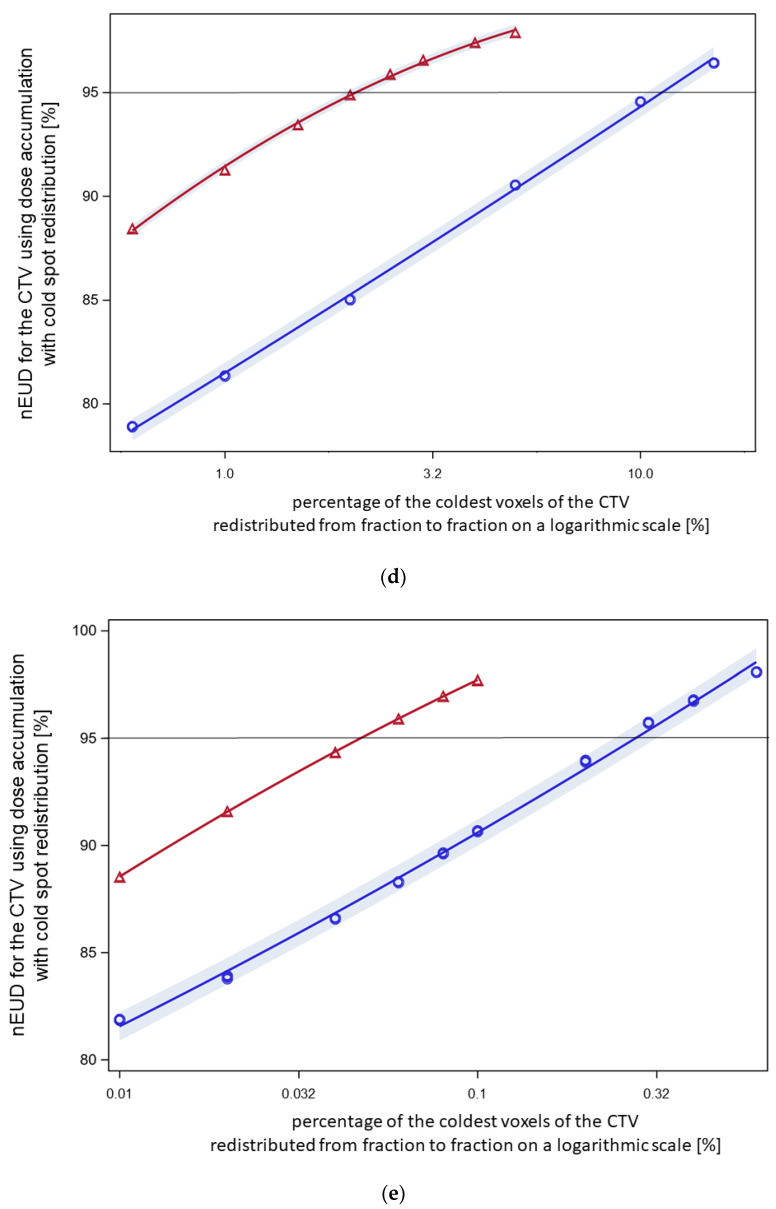
(**a**) Normalized EUD values for CTV of the nine focal treatment series using worst-case dose accumulation. Dose fractions were included for dose accumulation in order of nEUD_CTV_ from the fraction with the lowest to the highest nEUD_CTV_. Five series had nEUD_CTV_ values ≥ 95% (green symbols) while four series had an nEUD_CTV_ < 95% after the inclusion of a proportion nearest to 60% but ≥60% of dose fractions (blue symbols). Using worst-case accumulation across the whole series, the EUD values for the CTV ranged from 92.6 to 102.4%. (**b**) The effect of voxel redistribution from worst-case accumulation. The 60% of fractions with the lowest EUD values of this focal series (patient XA) were used, resulting in an nEUD of 94.3% with worst-case accumulation. Blue circles: the indicated percentages of the coldest voxels were redistributed within this cold spot (method 1). Therefore, redistribution was limited to the voxels with order numbers within this coldest part of the CTV. A redistribution rate of 0.6% was necessary with this mode of redistribution to raise the nEUD_CTV_ by accumulation to >95%. Red triangles: the indicated percentages of the coldest voxels per fraction were redistributed across the whole outer 5 mm rim of the CTV for all fractions except the fraction with the lowest nEUD_CTV_ (method 2). A linear quadratic relation was fitted to the logarithm of the percentage number of the redistributed coldest voxel to the base 10 as the independent variable. At a redistribution rate of 0.04% of the coldest voxels per fraction, the curve raised to nEUD_CTV_ values of >95%. (**c**) The effect of voxel redistribution by methods 1 and 2 for another focal radiotherapy series (patient D) with an nEUD_CTV_ of 82.9% using worst-case accumulation across 60% of fractions with the lowest nEUD_CTV_. Blue circles: A redistribution rate using method 1 of 2.0% was necessary to raise the nEUD to >95%. Red triangles: Redistribution of 0.4% of the coldest voxels per fraction from the 5 mm outer rim of the CTV resulted in nEUD values >95% using method 2. (**d**) See under Figure 1a. Worst-case accumulation resulted in an nEUD value of 70.5% for this third focal series (patient E). Blue circles: A redistribution rate of 15% was necessary for this radiotherapy series using mode 1 to raise the nEUD_CTV_ to >95%. Red triangles: Redistribution of 2.5% of the coldest voxels per fraction across the outer 5 mm rim of the CTV resulted in nEUD values >95%. (**e**) Worst-case accumulation resulted in a nEUD_CTV_ of 84.2% for this forth focal series (patient I). Blue circles: A redistribution rate of 0.3% was necessary using method 1 to raise the nEUD to >95%. Red triangles: Redistribution of 0.06% of the coldest voxels per fraction across the outer 5 mm rim of the CTV resulted in nEUD values >95%.

As dose accumulation with elastic deformation of the dose distributions between fractions appeared to be rather lenient for nEUD_CTV_ that declined below 80% of the prescribed dose for some dose fractions per series, we further studied this phenomenon using an independent method, not depending on elastic deformation, i.e., worst-case dose accumulation. Figure 4a shows the lower limits for the nEUD_CTV_ of the accumulated dose distributions for the nine focal bladder treatment series in dependence on an increasing proportion of dose fractions from the series included in dose accumulation. Thereby, the dose fractions per series were ordered according to their nEUD_CTV_, from the worst to the best nEUD_CTV_. Dose accumulation was started with five dose fractions from a series and was continued up to the inclusion of all fractions. After the inclusion of a proportion ≥60% of the dose fractions with the lowest nEUDCTV from a series, the nEUD_CTV_ using worst-case dose accumulation was above 95% of the prescribed dose for five series, while four series had nEUD_CTV_ values for the accumulated dose distribution between 70.5% and 94.3%. After the inclusion of 100% of the dose fractions from a series, worst-case dose accumulation demonstrated nEUD_CTV_ values > 95% in seven of the nine series, while two series with 9 and 13 fractions showed nEUD_CTV_ values of 94.9% and 92.6%. 

As a result, even with worst-case dose accumulation, the indicated proportion of nEUD_CTV_ values per series below 80% were tolerated and resulted in nEUD_CTV_ values under the accumulated dose distribution above 92.5%.

**Figure 5:**
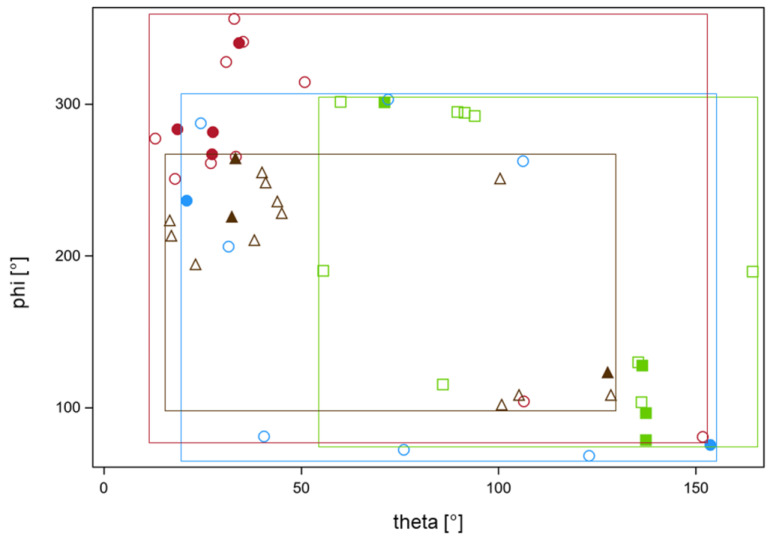
Locations of the dose minima on the surface of the CTV using the adaptive plans on CBCT2 for the four focal series detailed in Figure 4a failed to achieve a nEUD_CTV_ value > 95% at 60% of fractions as determined with worst-case dose accumulation. Locations of the minima were mapped on the planning CT for the respective series using deformable registration and were characterized by the polar and azimuth angles theta and phi in spherical coordinates with the geometric center of the respective CTV as the origin. A polar angle of 0° points points to the cranial (z) direction an azimuth angle of 0° in the x-direction. Each of the four series is indicated by a different color and symbols. Filled symbols indicate fractions with nEUD_CTV_ values < 0.76—the lower quartile of values from these four series. The rectangular frames indicate the ranges of values from the respective series labeled by the series color. The frames cover the majority of angles and indicate wide-spread minima. There were no differences in the polar or azimuth angles for the data points with foiled and open symbols (*p* > 0.5, Wilcoxon tests). There were significant inter-serial differences in phi and theta (*p* < 0.025, Kruskal–Wallis tests). The focal series labeled in red showed significant deviations form a uniform distribution of phi and theta (*p* < 0.001, Kolmogorov tests for both angles), while the series labeled in brown showed deviations in theta alone (*p* = 0.008, Kolmogorov test). The other angles from the other series were compatible with a uniform distribution.

In addition to worst-case dose accumulation, the effect of cold spot redistribution between fractions from worst-case accumulation was studied using two further methods: random permutation of a given percentage of the coldest voxels per fraction within these coldest voxels (method 1) and random permutation of a given percentage x of the coldest voxel across the whole 5 mm surface rim of the CTV (method 2). The results with both redistribution methods were shown at a percentage of included dose fractions nearest and ≥60% for the four series with nEUD_CTV_ values < 95% using worst-case dose accumulation was at this percentage (Figure 4b–e). Method 1 with limited redistribution among the coldest voxel showed lower nEUD_CTV_ values at a given percentage of redistributed voxels than the more widespread redistribution over the whole surface rim. The percentage of voxels needed to achieve an nEUD_CTV_ of 95% ranged between 0.28% to 11.3% of the coldest voxels for method 1 and from 0.03% to 2.1% for method 2. Figure 5 shows indeed, that wide distribution of the observed dose minima over the surface of the CTV took place from dose fraction to dose fraction for the four series. The location of the dose minima mapped on the CTV in the planning CT was indicated by spherical coordinates. There were some differences in phi and theta depending on the respective radiotherapy series (*p* < 0.025, Kruskal–Wallis tests). 

Worst-case accumulation with moderate redistribution of the coldest voxels was therefore justified from the observed spatial distribution of cold spots within a series and can fully explain the robustness of dose accumulation with elastic deformation to cold spots or low nEUD_CTV_ values in some of the dose fractions of a series.

**Figure 6:**
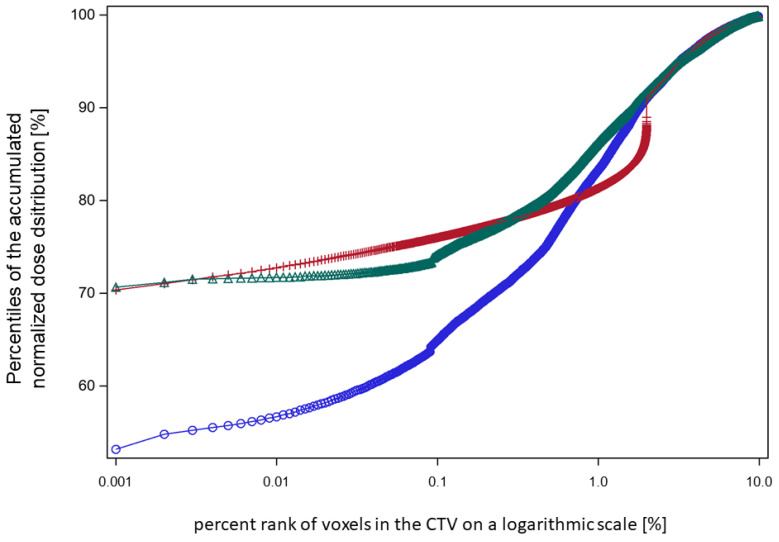
A plot of the lower percentiles of the accumulated dose distributions against the respective percent rank of the coldest voxels in the CTV at which the respective percentile was scored on a logarithmic scale (patient XD). Blue circles: worst-case dose accumulation of the 60% of fractions with the lowest nEUD_CTV_ values from the focal series shown in Figure 4c. Red crosses: random permutation of the 1.5% of the coldest voxels according to method 1. Green triangles: random redistribution of 0.4% coldest voxels per fraction across the whole outer 5 mm rim of the CTV according to method 2. The nEUD_CTV_ values for these accumulated dose distributions were: blue circles, 82.9%; red crosses, 95.5%; and green triangles, 95.9%.

In order to inspect the low-dose tail of the worst-case accumulated dose distribution and demonstrate the effect of redistribution by method 1 and method 2, we showed the dose percentile plots across the low-dose region for the series in Figure 4c, which includes 60% of the dose fractions (Figure 6). To stretch the low-dose range, the percentage numbers were provided in a logarithmic scale. With method 1, the coldest 1.5% of voxels, and with method 2, 0.4% of voxels, were redistributed—both sufficient to achieve an nEUD_CTV_ > 95%.

For comparison, we calculated the PTV margins with two conventional margin recipes, the method according to [36] and the margin growing method. Using the four treatment series from Figure 4a that had an nEUD_CTV_ < 95% with worst-case dose accumulation after inclusion of a proportion nearest to 60% of dose fractions, the required margin at the bladder dome in cranial direction was 11.5 mm for ART and 14.9 mm for IGRT to compensate intra- and inter-fractional deformations, respectively. For both ART and IGRT, the 95th percentiles of the cranial deviations of the global Hausdorff points were significantly larger than 5 mm in two of the four series. The margin-growing method showed that the required margins for ART in cranial and caudal directions were 15 mm and 12–18 mm in the other directions to encompass the CTV in ≥95% of dose fractions. Meanwhile, for IGRT, margins were 15 mm in the cranial direction, 10 mm in the caudal direction, as well as 12–26 mm in the other directions. The margin of >20 mm was in the anterior direction alone. In all directions, the 95th percentile for the outward deformation was significantly >5 mm in at least three of the four series. 

Thus, common margin recipes resulted in margins that were considerably larger than those found with worst-case or DIR-based dose accumulation.

**Figure 7:**
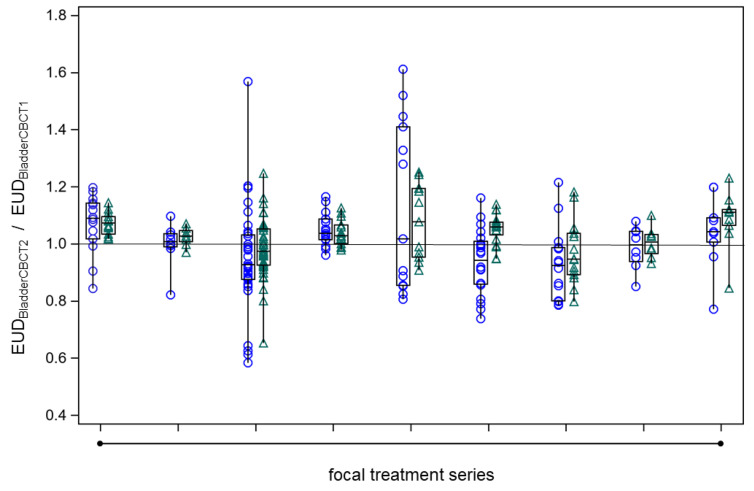
The constancy of bladder wall EUD outside the PTV on CBCT2 as determined by the adaptive plan (EUD_BladderCBCT2_) in comparison to the respective value on CBCT1 (EUD_BladderCBCT1_) across all fractions from the nine focal series. The boxes of the box plots represent the medians and the 25th or 75th percentiles for the different focal series. Blue circles: EUD_BladderCBCT2_ by the adaptive plan normalized to the respective EUD_BladderCBCT1_, generalized EUD determined with a = 1. Green triangles: same as under blue circles but with a = 8.

To assess the constancy of bladder sparing by the adaptive plans optimized on the PTV in CBCT1, we calculated EUD values for the bladder wall outside the PTV using model parameters of a = 1 and a = 8 spanning the range of previously determined values. Across all series, bladder sparing remained constant with ART on CBCT2 in comparison to CBCT1, whereby the EUD_BladderCBCT1_/EUD_BladderCBCT1_ ratios were not significantly different from unity (Figure 7, *p* = 0.10, signed rank test).

## 4. Discussion

Trimodality treatments, including transurethral resection of the bladder tumor, radiotherapy, and concurrent chemotherapy, are standard options for bladder preserving therapy of muscle-invasive bladder cancer. About 19% of patients receiving trimodality treatment require salvage cystectomy due to local recurrence or persistent tumor [41]. According to the results of the BC2002 trial, whole bladder radiotherapy—at an intermediate dose of 64 Gy in 32 fractions over 6.5 weeks or 55 Gy in 20 fractions over 4 weeks—could not improve the results in comparison to focal tumor-directed radiotherapy with a 1.5 cm PTV margin following whole bladder radiotherapy at 80% of the total dose [42]. In this dose range, similar acute and late urinary toxicities were observed using whole bladder radiotherapy or partial bladder radiotherapy as a boost [42,43]. However, according to the RTOG scale, the overall grade III-IV toxicity amounted to 16% in this trial [42].

With the increased availability of ART, there is rising interest in performing ART for bladder cancer to reduce these toxicities and to enable radiation dose escalation. Several studies on focal bladder tumor irradiation were performed with a full or half-full bladder [12,38,44,45]. Bladder volumes in the CBCT studies during the treatment sessions were smaller than bladder volumes in the planning CT in the study by Kong et al. (2019) [44], and the volume ratios ranged between 0.43 and 0.87 with a geometric mean value of 0.56. In the present study, we found a good reproducibility of bladder fillings for bladder volumes between 110 and 310 mL during treatment planning CT. The geometric mean ratio between bladder volume in the post-adaptation CT and bladder volume in planning CT was 0.77. 

Thus far, there were only two studies that reported on the feasibility of online ART [10,11]. Both studies treated patients with an empty bladder and included the whole bladder; in the case of the study by Aström et al. [10] elective lymph nodes were included into the CTV. In the latter study, patient-dependent PTV margins of 8–13 mm were used in the cranial direction and of 5–10 mm in the other directions, while in the study by Mitchell and co-workers [11], PTV margins of 15 mm anteriorly and superiorly and 5–10 mm in the other directions were used. In the present study, we used smaller individualized margins of 5–10 mm in cranial and anterior directions and 5–9 mm in the other directions. Therefore, the present study describes the results of focal bladder radiotherapy with online ART for the first time.

According to classical margin recipes, PTV margins calculated to cover inter-fractional positional errors in the cranial directions were 15–20 mm, while in the other directions, smaller margins of about 10–20 mm were used [1,37,38,39,46]. Margins of 5–10 mm were found to compensate for intra-fraction motion within 10 min in the cranial direction but the margins increased nearly linearly with time [47,48]. In the present study, we found that margins of about 15 mm in the cranial and 10–15 mm in the caudal direction according to the classical margin recipes compensated for inter- and intra-fractional deformations. Cranio-caudal margins were of high importance as the gradients of the dose distributions around the PTV were steeper in these directions than in the anterior-posterior or lateral directions with VMAT or IMRT techniques. However, the classical margin recipes did not account for the small size of the cold spots outside the 95% isodose line, nor the wider distribution of the cold spots across the surface of the CTV. This is over-conservative as shown here in comparison with worst-case accumulation which accounted for the small size of the cold spots or the DIR-based dose accumulation, which accounted for the small size and the positional variation of cold spots fully. Through DIR-based dose accumulation or worst-case dose accumulation with reordering of cold spots in this study, we found that margins of 5–10 mm were sufficient to compensate intra-fraction deformation using ART with median adaptation times of 18.8 min for all of the nine focal radiotherapy series.

The degree of correlation of the location of cold spots at the margin of the CTV between fractions and thereby the randomness of the occurring intra-fractional deformations can be assessed by the comparison of the nEUD values obtained with the worst-case or DIR-based dose accumulation. The direct location of the dose minima showed a wide spread over the surface of the CTV in this study. Dose accumulation determined from the voxel order number of the dose–volume histograms—which were initially sorted by their increasing dose values using additional reordering algorithms for the voxels in the low-dose tail—is a tool to assess the coupling of cold spots between fractions. With these tools, predictions can be made about the expected EUD of the accumulated dose distribution at the end of a radiation series from the fractions, which were delivered up to now during the course of treatment. Risks that occur at the tails of multivariate distributions, here the low-dose regions of the differential dose-volume histograms from different dose fractions, are studied in different fields, such as finance economics [49] or computational risk management [50].

In this study, we showed that worst-case dose accumulation can be a sensitive instrument for detecting during treatment if and when the accumulated EUD for the CTV undercuts a critical limit. This event may trigger a switch from IGRT towards ART. Worst-case dose accumulation also requires the creation of the CTV on the day of the pretreatment with CBCT or MRI as DIR-based dose accumulation. This can be obtained using several methods such as hybrid intensity or marker-based deformation of the reference anatomy on the CBCT [51,52], or use of artificial intelligence-generated normal tissue contours to create the target volume of the day with deformable registration using the auto-contoured normal tissue contours [53], or deep learning models based on convolutional neural networks [54]. However, worst-case accumulation avoids some errors that can arise from wrapping the deformed CTV of the day on reference anatomy. The created CTV of the day can deviate from the intended CTV. Influencer structures of the CTV of the bladder or rectum generated with different DIR may show considerable variations between algorithms and may differ from manually delineated structures [52,55]. It seems to be important for cold spots with worst-case accumulation to be sensitive, given the marked robustness of DIR-based dose accumulation against EUD declines in the 10th percentile of the EUD_CTV_ values obtained from the dose fractions of a series.

Algorithms, that can automatically assess whether IGRT is sufficient for the considered dose fraction or treatment series or whether online ART will have advantages, are of great interest [16]. Lim et al. analyzed the Jacobian Matrix obtained from deformable image registration of the planning CT onto the daily prefraction CBCT and identified parameters, that indicated an advantage of ART over IGRT [56]. Such results could be improved using convolutional neural networks for auto-contouring of the prefraction imaging studies [54,57]. In the present study, we showed that selective ART using ART only for fractions with worse EUD values of below 93% from IGRT yields an equivalent EUD for the accumulated dose distribution as continuous ART across the whole series.

The PTV margins of 5–10 mm used in this study could maintain the nEUD_CTV_ from the accumulated dose distribution above 95% for all series using DIR-based dose accumulation or worst-case dose accumulation with limited redistribution of cold spots. Such smaller margins are necessary for focal precision radiotherapy of localized bladder cancers as bladder volumes above 310 mL during planning were found to be not reproducible during the treatment series. Conventional PTV margins of 1.5 cm to 2.0 cm can significantly diminish the bladder wall outside the PTV, which can be spared by ART by 25% or more, depending on the bladder geometry and shape of the CTV. Using PTV margins from 5 to 10 mm and ART for dose fractions with low nEUD_CTV_ with IGRT can make focal bladder tumors more flexible and potentially more tumor selective than focal radiotherapy with a preformed plan library of three treatment plans per patient used in a phase II randomized controlled trial with dose escalation [12]. In addition, PTV margins for the main dose fractions can be individualized from the first few dose fractions of a treatment series with the methods introduced in this study as worst-case dose accumulation with redistribution. Beneath such dosimetric optimizations, precision radiation oncology relies on the accuracy of gross tumor volume definition as well as biomarkers for tumor and normal tissue responsiveness [58]. For bladder cancer, high-resolution micro-ultrasound is a promising new method that can help gross tumor volume definition complementary to cystoscopy with TRUB [59]. Urinary microRNAs are associated with bladder cancer aggressiveness and should be validated as biomarkers in further studies [60].

This study is not a trial with a sufficient follow-up that allows a comparison of ART with small PTV margins to IGRT at clinical end points such as survival or local control of invasive bladder cancer. However, this study used the nEUD_CTV_ values as a dosimetric endpoint and dosimetric endpoints are very sensitive for tumors following steep dose–response relations [61,62]. Tumor localisations across the bladder differed between patients in this study and bladder cancer can influence intra-fraction bladder wall deformations within the involved localization [63]. The small group of patients prevented a subgroup analysis according to tumor localization. In addition, adaptation times varied over a wider range in this study as both VMAT and 12-field IMRT were used. With 12-field IMRT adaptation, times can be reduced by about 10 min allowing less time for intra-fraction deformations [28]. This study did not allow a validated prediction of the individual minimum PTV margin for a bladder cancer patient, which was sufficient to compensate for intra-fraction deformation during dose adaptation so farhowever, it introduced worst-case dose accumulation with limited redistribution of cold spots to predict the dosimetric outcome of a whole treatment series using the first few fractions of the series, and to monitor the effectiveness of the delivered dose fractions thus far from the dose–volume histograms for the CTV or organs at risk of these fractions alone without the recourse to DIR-based dose accumulation.

## 5. Conclusions

ART showed a good dose coverage of the CTV over a treatment series, applying ≥8 dose fractions in the focal radiotherapy of bladder cancer with a full bladder at PTV margins of 5–10 mm. This was determined by both DIR-based and worst-case dose accumulation with limited redistribution of cold spots due to the small size and spatial variation of cold spots. Dose accumulation resulted in smaller PTV margins than classical margin recipes using geometric deformations alone. Selective use of ART only for dose fractions with larger nEUD_CTV_ declines with IGRT is supported by these data. Prospective trials using ART at small PTV margins are warranted, preferably using online dose accumulation in the CTV on post-adaptation imaging shortly after each dose fraction.

## Data Availability

The datasets generated and analyzed during the current study are not publicly available due to the right of personal data protection but can be made available by the corresponding authors upon reasonable request.

## Abbreviations

Adaptive radiotherapy (ART); clinical target volume (CTV); computed tomography (CT); cone-beam computed tomography (CBCT); confidence interval (CI); deformable image registration (DIR); dose-volume histogram (DVH); equivalent uniform dose (EUD); image-guided radiotherapy (IGRT); magnetic resonance imaging (MRI); normalized equivalent uniform CTV dose (nEUD_CTV_); planning target volume (PTV); transurethral resection of bladder tumor (TURBT).
